# Resveratrol attenuates constitutive STAT3 and STAT5 activation through induction of PTPε and SHP-2 tyrosine phosphatases and potentiates sorafenib-induced apoptosis in renal cell carcinoma

**DOI:** 10.1186/s12882-016-0233-7

**Published:** 2016-02-25

**Authors:** Chulwon Kim, Sang Hyun Baek, Jae-Young Um, Bum Sang Shim, Kwang Seok Ahn

**Affiliations:** Department of Science in Korean Medicine, College of Korean Medicine, Kyung Hee University, 24 Kyungheedae-ro, Dongdaemungu, Seoul, 130-701 Republic of Korea

**Keywords:** Resveratrol, STAT3/5, PTPε, SHP-2, Renal cell carcinoma

## Abstract

**Background:**

Signal transducers and activators of transcription (STAT) proteins are critical transcription factor that are aberrantly activated in various types of malignancies, including renal cell carcinoma (RCC).

**Methods:**

We investigated the effect of resveratrol (RES), an edible polyphenol phytoalexin on STAT3 and STAT5 activation cascade in both Caki-1 and 786-O RCC cell lines.

**Results:**

We found that RES suppressed both constitutive STAT3 (tyrosine residue 705 and serine residue 727) and STAT5 (tyrosine residue 694 and 699) activation, which correlated with the suppression of the upstream kinases (JAK1, JAK2, and c-Src) in RCC. Also, RES abrogated DNA binding capacity and nuclear translocation of these two transcription factors. RES-induced an increased expression of PTPε and SHP-2 and the deletion of these two genes by small interfering RNA abolished the ability of RES to inhibit STAT3 activation, suggesting the critical role of both PTPε and SHP-2 in its possible mechanism of action. Moreover, RES induced S phase cell cycle arrest, caused induction of apoptosis, loss of mitochondrial membrane potential, and suppressed colony formation in RCC. We also found that RES downregulated the expression of STAT3/5-regulated antiapoptotic, proliferative, and metastatic gene products; and this correlated with induction of caspase-3 activation and anti-invasive activity. Beside, RES potentiated sorafenib induced inhibitory effect on constitutive STAT3 and STAT5 phosphorylation, apoptotic effects in 786-O cells, and this correlated with down-regulation of various oncogenic gene products.

**Conclusion:**

Overall, our results suggest that RES is a blocker of both STAT3 and STAT5 activation and thus may exert potential growth inhibitory effects against RCC cells.

## Background

Signal transducer and activator of transcription (STAT) proteins initially discovered as latent cytoplasmic transcription factors about two decades ago [1], consist of seven diverse members,STAT1 to STAT6, STAT5a and STAT5b [2], that have been found to play a pivotal role in regulating inflammation and tumorigenesis [3]. Among the different STATs, STAT3 and STAT5 are often constitutively active in various human cancers, including renal cell carcinoma (RCC) [4–6], and control the expression of multiple genes involved in initiation, progression and chemoresistance [7–9]. Once STATs are activated, it translocates into the nucleus and controls the transcription of various oncogenic genes [3]. The phosphorylation is mediated through the activation of various upstream kinases called Janus activated kinases (JAKs) [1, 10]. The activation of STAT3 can induce the expression of various gene products required for apoptosis inhibitors (bcl-xl, bcl-2, IAP-1/2, and survivin), cell-cycle regulators (cyclin D1, and cyclin E), and inducers of angiogenesis (MMP-9 and VEGF) [11, 12]. Like STAT3, STAT5 has been also shown to regulate proliferation and inhibition of apoptosis in several cancer cells because cyclin D1 and bcl-xL promoters contain putative STAT5 binding sites [13, 14]. Thus, identification of novel pharmacological agents which inhibit STAT3 and STAT5 activation have promise and potential in prevention and therapy of cancer [15].

The identification of novel anticancer agents derived from Mother nature offer a great opportunity to improve the existing standard of care for RCC and other cancers [16]. RES (trans 3, 4’, 5-trihydroxystilbene) is a naturally occurring polyphenol phytoalexin and can be found in approximately 72 plant species, including grapes, peanuts, red wine and weed *Polygonumcuspidatum* [17–20]*.* In plants, RES functions microbiologically as a phytoalexin that protects against fungal infections [21, 22]. Preclinical studies demonstrate that RES has been found to be effective against various types of human cancers [23]. In addition, previous studies documented it has the ability to affect tumor initiation and promotion, inhibit angiogenesis and metastasis, and induce cell cycle arrest and apoptosis [24–26].

Renal cell carcinoma (RCC) is the most common malignancy of the adult kidney, and the incidence of newly diagnosed renal cell carcinoma cases have been steadily increasing over two decades [27–29]. Unlike many other cancers, there are few biomarkers and prognosis for RCC [30], and renal cancer patients display resistance to both conventional therapy and radiation treatment [31–33].

Hence, the discovery of novel therapeutics or molecular targeted therapies for RCC remains a priority. Previous reports show high frequency of increased STATs activation in RCC cells and patient specimens [4, 34, 35]. Because of the pivotal role of STATs in tumor cell survival, proliferation, and angiogenesis, we hypothesized that STAT3 and STAT5 could be a novel therapeutic target for RCC. Thus, in our study, we examined whether RES can exert its anticancer effects by negative regulation of STAT3/5 signaling cascade.

## Methods

### Reagents

Resveratrol (RES), 3-(4,5-dimethylthiazol-2-yl)-2,5-diphenyltetrazolium bromide (MTT), propidium iodide (PI), Tris base, glycine, NaCl, sodium dodecylsulfate (SDS), and bovine serum albumin (BSA) were purchased from Sigma-Aldrich (St. Louis, MO). RPMI 1640, fetal bovine serum (FBS), antibiotic-antimycotic mixture, and LightShift® Chemiluminescent EMSA kit were obtained from Thermo Fisher Scientific Inc. (Waltham, MA). 5’-biotinylated STAT3 and STAT5 was from Bioneer Corporation (Daejeon, Korea). Alexa Fluor® 488 donkey anti-rabbit IgG (H + L) antibody, and 0.4 % trypan blue vital stain, and TMRE (tetramethylrhodamine, ethyl ester) were obtained from Life Technologies (Grand Island, NY). Anti-phospho-STAT3(Tyr705), anti-phospho-STAT3(Ser727), anti-phospho-JAK1(Tyr1022/1023), anti-JAK1, anti-phospho-JAK2(Tyr1007/1008), anti-JAK2, and anti-phospho-Src(Tyr416) antibodies were purchased from Cell Signaling Technology (Beverly, MA). Anti-STAT3, anti-phospho-STAT5(Tyr 694/Tyr 699), anti-STAT5, anti-Src, anti-PTPε, anti-SHP-2, anti-bcl-2, anti-bcl-xL, anti-survivin, anti-IAP-1, anti-IAP-2, anti-COX-2, anti-VEGF, anti-MMP-9 (matrix metalloproteinase-9), anti-caspase-3, anti-cleaved caspase-3, anti-PARP, anti-cyclin D1, anti-cyclin E, anti-Bax, anti-p21, anti-p53, anti-β-actin, and horseradish peroxidase (HRP)-conjugated secondary antibodies were obtained from Santa Cruz Biotechnology (Santa Cruz, CA). Annexin V staining kits (ApoScan) were purchased from BioBud (Seoul, Korea). TUNEL (terminal transferase mediated dUTP-fluorescein nick end labeling) assay kit was from Roche Diagnostics GmbH (Mannheim, Germany).

### Cell lines

Human Renal cell carcinoma Caki-1 and 786-O were obtained from the American Type Culture Collection (Manassas, VA). Caki-1 and 786-O cells were cultured in RPMI 1640 medium containing 10 % FBS. Media were also supplemented with 100 U/ml of penicillin and 100 μg/ml of streptomycin.

### Western blotting

Western blot analysis was performed using a method described previously [36].

### EMSA for STAT3 and STAT5-DNA binding

Electrophoretic mobility shift assay (EMSA) was performed as described previously [36]. The membrane was detected following manufacturer instructions using LightShift® Chemiluminescent EMSA kit (Waltham, MA).

### Immunocytochemistry for STAT3 and STAT5 localization

Immunocytochemistry was performed as described previously [37].

### Reverse transcription polymerase chain reaction (RT-PCR)

Reverse transcription polymerase chain reaction was performed using a method described previously [38].

### Transfection with PTPε and SHP-2 siRNA

Caki-1 and 786-O cells were plated in 6-well plates and allowed to adhere for overnight incubation. On the day of transfection, 6 μl of Lipofectamin 2000 (Invitrogen, Carlsbad, CA) were added to 50 nM PTPε and SHP-2 siRNA in a final volume of 1 ml culture medium. After 5 h of transfection, cells were treated with RES for 8 h and whole-cell extracts were prepared for PTPε, SHP-2, phospho-STAT3, and STAT3 analysis by Western blot analysis.

### Cell cycle analysis

Cell cycle analysis was performed as described previously [37].

### Annexin V assay

Annexin V assay was performed using a method described previously [37]

### Measurement of mitochondrial membrane potential

Mitochondrial membrane potential assay was performed using a method described previously [37].

### Clonogenic assay

Clonogenic assay was performed as described previously [39].

### Monitoring of cell growth with the RTCA DP instrument

Real-time cell proliferation analysis assay was performed using a method described previously [39].

### Invasion assay

Invasion assay was performed as described previously [37].

### MTT assay

Cell viability was measured by an MTT assay to detect NADH-dependent dehydrogenase activity [40], as described previously [39].

### Analysis of drug interactions

Analysis of drug interactions were performed using a method described previously [41]

### Statistical analysis

Statistical analysis was performed by Student’s *t*-test and one way analysis of variance, (ANOVA). A *p* value of less than 0.05 was considered statistically significant.

## Results

### RES down-regulates constitutive STAT3 and STAT5 phosphorylation in renal cell carcinoma

**Fig. 1:**
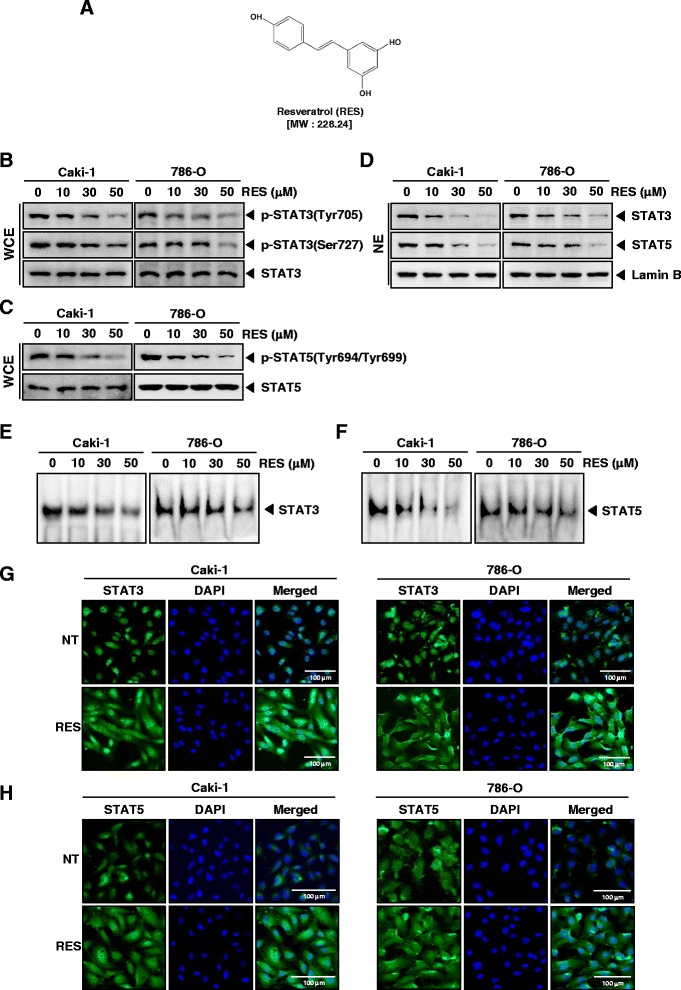
RES suppresses phosphorylation of STAT3 and STAT5 in a dose-dependent manner in RCC cells. **a** The chemical structure of resveratrol (RES). **b** and **c** Caki-1 and 786-O cells (1 × 10^6^ cells/well) were incubated at 37 °C with various indicated concentrations of RES for 6 h. Whole-cell extracts were prepared, then equal amounts of lysates were analyzed by Western blot analysis using antibodies against p-STAT3(Tyr705), p-STAT3(Ser727), STAT3, p-STAT5(Tyr694/Tyr699), and STAT5. The results shown here are representative of three independent experiments. **d** Caki-1 and 786-O cells (1 × 10^6^ cells/well) were incubated at 37 °C with various indicated concentrations of RES for 6 h. After that, nuclear proteins were extract, equal amounts of lysates were analyzed by Western blot analysis using antibodies against STAT3 and STAT5. The same blots were stripped and reprobed with Lamin B antibody to verify equal protein loading. The results shown here are representative of three independent experiments. **e** and **f** RES suppresses STAT3 and STAT5 binding activity in Caki-1 and 786-O cells. After Caki-1 and 786-O cells (1 × 10^6^ cells/well) were incubated at 37 °C with the indicated concentrations of RES for 6 h, analyzed for nuclear STAT3 and STAT5 levels by EMSA. The results shown here are representative of three independent experiments. **g** and **h** RES causes inhibition of translocation of STAT3 and STAT5 to the nucleus. Caki-1 and 786-O cells (3 × 10^4^ cells/well) were incubated at 37 °C with 50 μM of RES treatment, the cells were fixed and permeabilized. STAT3 and STAT5 (green) was immunostained with rabbit anti-STAT3 and anti-STAT5 followed by FITC-conjugated secondary antibodies and the nuclei (blue) were stained with DAPI. The third panels show the merged images of the first and second panels. Scale bar = 100 μm. The results shown are representative of two independent experiments

We first tested whether RES (structure of resveratrol, Fig. 1a) inactivated STAT3 in Caki-1 and 786-O renal cancer cells. Previous studies have shown that STAT3 is a key point of convergence of multiple oncogenic signaling pathways [42–44]. The ability of RES to modulate constitutive STAT3 activation in RCC cells was investigated by Western blot analysis using antibodies which recognize STAT3 phosphorylation at tyrosine 705 and serine 727. As shown in Fig.1b, RES inhibited the constitutive activation of STAT3 in both Caki-1 and 786-O cells in a dose-dependent manner, with maximum inhibition occurring at around 50 μM, but had no effect on the expression of STAT3 protein (Fig. 1b, *third panels*). Whether RES affects the activation of another STAT family isoform, STAT5 in renal cancer cells was also investigated. We noticed that RES substantially reduced constitutive STAT5 activation in both Caki-1 and 786-O cells, without affecting total STAT5 levels as analyzed by Western blot analysis (Fig. 1c).

### RES reduces STAT3 and STAT5 in nuclei

We tested the effect of RES on STAT3 and STAT5 in nuclei. As shown by Western blot analysis in Fig. 1d, RES inhibits nuclear STAT3 and STAT5 in Caki-1 and 786-O cells.

### RES inhibits binding capacity of STAT3 and STAT5 to the DNA

We next determined whether RES can modulate the DNA-binding ability of STAT3 and STAT5 proteins in RCC cells. EMSA analysis of nuclear extracts prepared from Caki-1 and 786-O cells showed that RES reduced STAT3 and STAT5-DNA binding activities in a dose-dependent manner (Fig. 1e and f). These results show that RES abrogates the DNA binding ability of STAT3 and STAT5.

### RES reduces nuclear pool of STAT3 and STAT5 in renal cell carcinoma

We next analyzed whether RES can suppress nuclear translocation of STAT3 and STAT5 in RCC cells. Figure 1g and h clearly demonstrates that RES reduced the translocation of STAT3 and STAT5 to the nucleus in both Caki-1 and 786-O cells.

### RES inhibits activation of upstream kinases involved in STAT3/5 signaling cascade in RCC cells

**Fig. 2:**
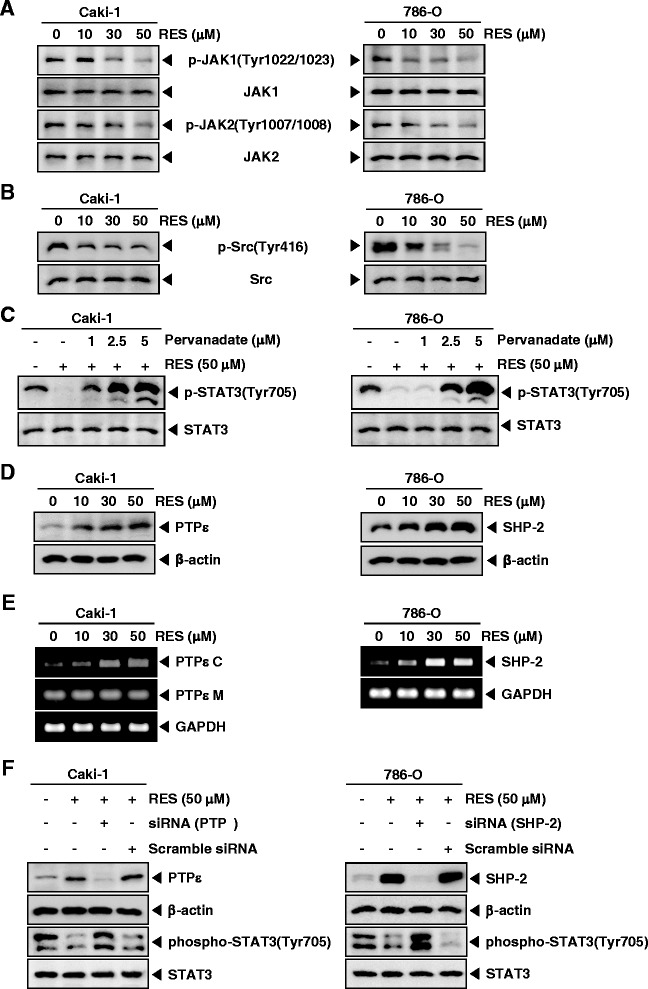
Pervanadate reverses the phospho-STAT3 inhibitory effect of RES. **a** and **b** Caki-1 and 786-O cells (1 × 10^6^ cells/well) were incubated at 37 °C with various indicated concentrations of RES for 6 h. Whole-cell extracts were prepared, then equal amounts of lysates were analyzed by Western blot analysis using antibodies against p-JAK1(Tyr1022/1023), JAK1, p-JAK2(Tyr1007/1008), JAK2, p-Src(Tyr416), and Src. The results shown here are representative of three independent experiments. **c** Pervanadate reverses the p-STAT3 inhibitory effect of RES. Caki-1 and 786-O cells (1 × 10^6^ cells/well) were co-incubated with the indicated concentrations of pervanadate and 50 μM RES for 6 h, after which whole-cell extracts were prepared and 15 μg portions of those extracts were resolved on 8 % SDS-PAGE gel, electrotransferred onto nitrocellulose membranes, and probed for p-STAT3(Tyr705) and STAT3. **d** Caki-1 and 786-O cells (1 × 10^6^ cells/well) were treated with various indicated concentrations of RES for 6 h, after which whole-cell extracts were prepared and 10 μg portions of those extracts were resolved on 8 % SDS-PAGE, electrotransferred onto nitrocellulose membranes, probed for PTPε and SHP-2 antibody. The same blots were stripped and reprobed with β-actin antibody to verify equal protein loading. **e** Caki-1 and 786-O cells (1 × 10^6^ cells/well) were treated with various indicated concentrations of RES for 6 h, and total RNA was extracted and examined for expression of PTPε C, PTPε M, and SHP-2 by RT-PCR. GAPDH was used as an internal control to show equal RNA loading. **f** Effect of PTPε knockdown on RES induced expression of PTPε. Caki-1 cells were transfected with either PTPε siRNA or scrambled siRNA (50 nM). After 48 h, cells were treated with 50 μM RES for 6 h and whole-cell extracts were subjected to Western blot analysis (*left panels*). Effect of SHP-2 knockdown on RES induced expression of SHP-2. 786-O cells were transfected with either SHP-2 siRNA or scrambled siRNA (50 nM). After 48 h, cells were treated with 50 μM RES for 6 h and whole-cell extracts were subjected to Western blot analysis (*right panels*)

In order to determine which upstream signaling molecules are involved in RES-mediated STAT3/5 inactivation, we examined the effects of RES on the phosphorylation of JAK1, JAK2, and Src in Caki-1 and 786-O cells. Cells were treated with the indicated concentrations of RES for 6 h. As shown in Fig. 2a, JAK1 and JAK2 were constitutively active in Caki-1 and 786-O cells and the treatment with RES clearly suppressed its phosphorylation in a concentration-dependent manner. The levels of total JAK1/2 remained unchanged under the same conditions (Fig. 2a, *second and fourth panels*).

We also determined the effect of RES on constitutive activation of Src kinase in both Caki-1 and 786-O cells. Interestingly, RES suppressed the constitutive phosphorylation of Src kinase in a concentration-dependent manner in these cells (Fig. 2b).

### Tyrosine phosphatases are involved in RES-induced inhibition of STAT3 activation

To analyze whether RES-induced inhibition of STAT3 phosphorylation could be due to activation of a protein tyrosine phosphatase (PTPase), Caki-1 and 786-O cells were treated with the broad-acting tyrosine phosphatase inhibitor sodium pervanadate. We noted that prevented the RES-induced inhibition of STAT3 activation (Fig. 2c). This data suggests that tyrosine phosphatases may be involved in RES-induced inhibition of STAT3 activation in RCC cells.

### RES induces the expression of PTPε and SHP-2 in RCC cells

Protein tyrosine phosphatases have been also implicated in the STATs signaling pathways [45]. Thus, we examined whether RES regulates the expression of PTPε, and SHP-2, which are non-transmembrane PTPs expressed most abundantly in hematopoietic cells [36, 37, 46, 47]. As shown in Fig. 2d, RES led to increased expression of PTPε in Caki-1 cells, and SHP-2 in 786-O at the protein level. RES also enhanced mRNA level of PTPε C and SHP-2 in a dose dependent manner in Caki-1 and 786-O cells (Fig. 4e).

### PTPε siRNA down-regulate the expression of PTPε and reverses the inhibition of STAT3 activation by RES in Caki-1 cells

We determined whether the suppression of PTPε expression by siRNA would abrogate the inhibitory effect of RES on STAT3 activation in Caki-1 cells. Western blotting showed that RES-induced PTPε expression was effectively abolished in the cells treated with PTPε siRNA; whereas treatment with scrambled siRNA had no effect (Fig.2f, *left, first panel*). We also found that RES did not suppress STAT3 activation in cells treated with PTPε siRNA (Fig. 2f, *left, third panel*).

### SHP-2 siRNA down-regulate the expression of SHP-2 and reverses the inhibition of STAT3 activation by RES in 786-O cells

We also determined whether the deletion of SHP-2 expression by siRNA would abolish the suppressive effect of RES on STAT3 activation in 786-O cells. We found that RES-induced SHP-2 expression was effectively prevented in the cells treated with SHP-2 siRNA; treatment with scrambled siRNA had no effect (Fig. 2f, *right, first panel*). RES did not suppress STAT3 activation in cells treated with SHP-2 siRNA (Fig. 2f, *right, third panel*).

### RES induces S phase cell cycle arrest in renal cell carcinoma

**Fig. 3:**
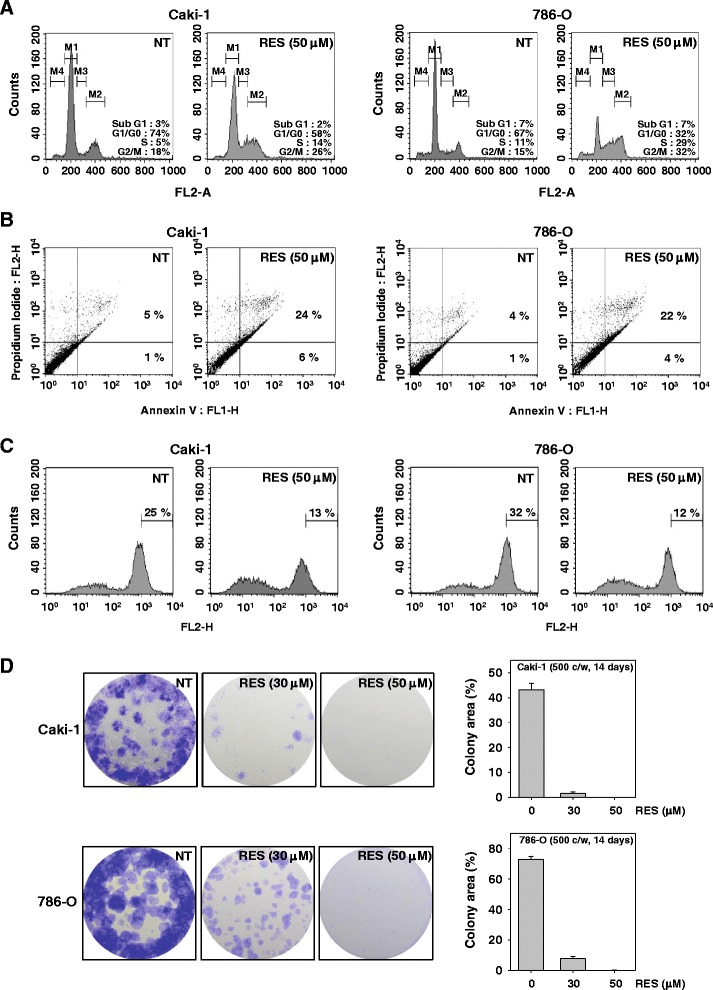
RES causes the accumulation of cells in S phase, induces apoptosis, and suppresses colony forming ability in renal cell carcinoma. **a** Caki-1 and 786-O cells (1 × 10^6^ cells/well) were treated with 50 μM of RES for 24 h. Then, the cells were fixed and analyzed using a flow cytometry. The results shown here are representative of three independent experiments. **b** Caki-1 and 786-O cells (1 × 10^6^ cells/well) were treated with 50 μM of RES for 24 h. Afterward, the cells were incubated with anti-Annexin V antibody conjugated with FITC plus PI and analyzed with a flow cytometer for apoptotic effects. The results shown here are representative of three independent experiments. **c** Two cells (1 × 10^6^ cells/well) were treated with RES at 50 μM concentrations for 24 h and the cells were incubated with TMRE (tetramethylrhodamine, ethyl ester) and then analyzed by a flow cytometry. The results shown here are representative of three independent experiments. **d** Caki-1 and 786-O cells were incubated with RES and subsequently allowed to grow into colonies. After 14 days incubation, cells were fixed with 10 % formalin and stained with crystal violet reagent, photographed and colonies were counted and represented graphically. Results are representative of two independent experiments

We also examined the effects of RES on cell cycle progression in Caki-1 and 786-O cells. As shown in Fig. 3a, RES-induced an increased accumulation of the cell population in S phase and a corresponding decrease of cells in the G1/G0 phases on Caki-1 and 786-O cells.

### RES promotes substantial apoptosis in renal cell carcinoma

To evaluate the anti-cancer effects of RES, we also examined the apoptosis-inducing effects of RES by using the Annexin V assay. The Annexin V positive cells were increased as compared with the non-treated cells and Annexin V-FITC and PI positive cells were also increased in Caki-1 and 786-O cells (Fig. 3b).

### RES causes loss of mitochondrial membrane potential in RCC cells

We also examined whether RES induces changes in the mitochondrial membrane potential in Caki-1 and 786-Ocells. TMRE is a cell permeable, positively-charged, red-orange dye that readily accumulates in active mitochondria due to their relative negative charge. Depolarized or inactive mitochondria have decreased membrane potential and fail to sequester TMRE. As shown in Fig. 3c, the accumulation of TMRE labeled mitochondria was notably reduced on RES treatment for 24 h as compared with the non-treated cells. These results suggest that RES induces both early and late apoptosis accompanied with mitochondrial dysfunction in these RCC cells.

### RES suppresses colony formation of RCC cells

A clonogenic assay evaluates the potential of a single cell to resist treatments and grow into a colony [48]. The influence of RES on the clonogenic capacity of Caki-1 and 786-O cells was also evaluated. RES significantly inhibited colony formation and resulted in a remarkable decrease at colony formation ratio (Fig. 3d).

### RES represses the expression of various proteins involved in anti-apoptosis, proliferation, and angiogenesis

**Fig. 4:**
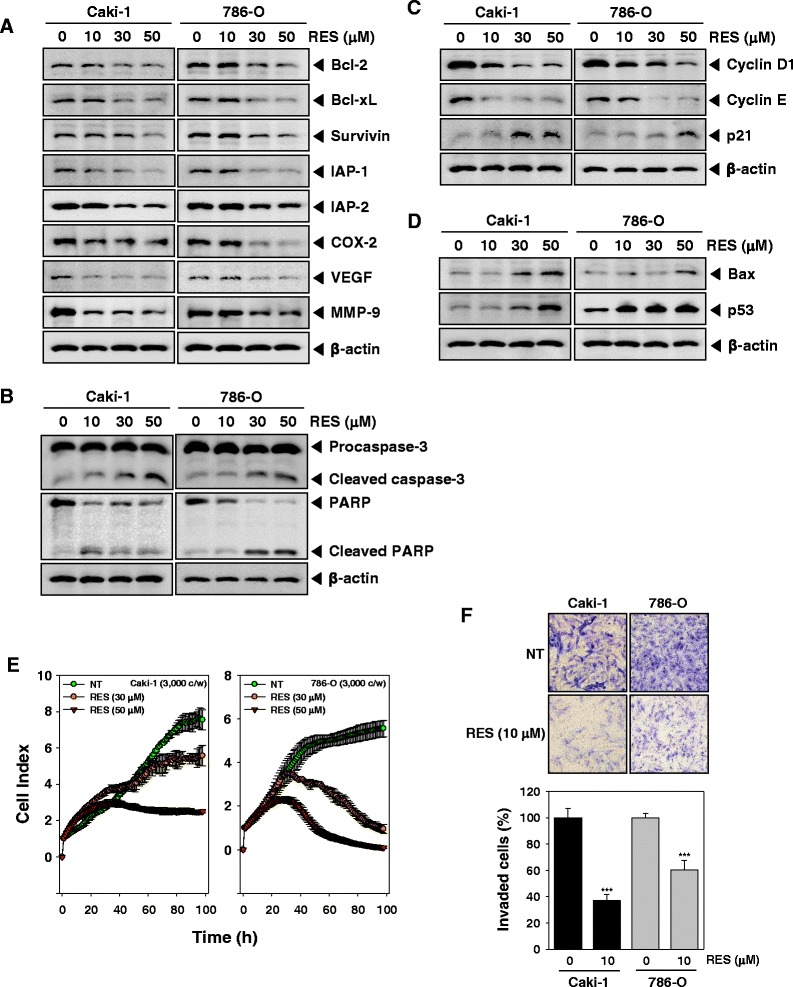
RES suppresses expression of various proteins involved in anti-apoptosis, proliferation, and angiogenesis. **a**-**d** After Caki-1 and 786-O cells (1 × 10^6^ cells/well) were incubated with the indicated various concentrations of RES for 24 h. Whole-cell extracts were prepared, and 20 μg of the whole-cell lysate was resolved by SDS-PAGE, electrotransferred to nitrocellulose membrane, and then probed with antibodies against bcl-2, bcl-xL, survivin, IAP1/2, COX-2, VEGF, MMP-9, caspase-3, PARP, cyclin D1, cyclin E, p21, Bax, and p53 as described in “Materials and methods.” The same blots were stripped and reprobed with β-actin antibody to verify equal protein loading. The results shown here are representative of three independent experiments. **e** Cell proliferation assay was performed using the Roche xCELLigence Real-Time Cell Analyzer (RTCA) DP instrument (Roche Diagnostics GmbH, Germany) as described in “Material and methods”. After Caki-1 and 786-O cells (5 × 10^3^ cells/well) were seeded onto 16-well E-plates and continuously monitored using impedance technology. **f** Caki-1 and 786-O cells were added to the upper side of the invasion chamber, the cells on the upper surface of the filter were removed after 24 h of incubation in the presence or absence of 10 μM of RES. The cells on the lower surface were fixed, stained and monitored by photographing, then counted. Randomly chosen fields were photographed under a light microscope at 100× magnification and invaded cells were counted

Because bcl-2, bcl-xL, survivin, IAP-1, and IAP-2 have been implicated in apoptosis and mitochondrial dysfunction, we next examined the effects of RES on the constitutive expression of these proteins by Western blot analysis. Cells were treated with 10, 30, or 50 μM of RES for 24 h. We observed that the constitutive expression of bcl-xl, bcl-2, survivin, IAP-1, and IAP-2 was dose-dependently reduced in RES-treated cells. Also, RES suppressed proteins linked with cell proliferation (COX-2), and angiogenesis (VEGF, and MMP-9) (Fig. 4a).

### RES activates caspase-3 and causes PARP cleavage

Whether suppression of constitutively active STAT3 in Caki-1 and 786-O cells by RES leads to apoptosis was also investigated. In these RCC cells treated with RES there was a dose-dependent activation of pro-caspase-3 and increased expression of cleaved caspase-3. Caki-1 and 786-O cells were treated with indicated concentration of RES for the 24 h, and then examined for caspase activation by Western blot using specific antibodies. We found a dose-dependent activation of caspase-3 by RES in these RCC cell lines (Fig. 4b, *first panel*). Activation of downstream caspases led to the cleavage of a 116 kDa PARP protein into 87 kDa fragments (Fig. 4b, *Second panel*). Taken together, these results suggest that RES induces caspase-3-dependent apoptosis in Caki-1 and 786-Ocells.

### RES alters the expression of cell cycle-associated proteins

We also investigated whether RES blocks expression of various cell cycle associated proteins. As shown in Fig. 4c, RES induced a dose-dependent decrease in cyclin D1 and cyclin E protein levels, while an increased expression of p21 was observed after RES treatment (Fig. 4c, *third panel*). Taken together, these results indicate that RES decreased growth by blocking cell cycle progression via the upregulation of p21 and/or the downregulation of cyclin D1 and cyclin E expression.

### RES induces the expression of both Bax and p53 in RCC cells

To determine whether RES induces expression of Bax and p53, we examined the expression of these proteins in Caki-1 and 786-O cells by Western blot analysis. Figure 4d shows that RES induced the expression of Bax and p53 gene products in a dose-dependent manner.

### RES significantly suppresses the viability of RCC cells

To specifically examine the anti-tumor activity of RES on Caki-1 and 786-O cells, the cells were treated with 30 or 50 μM concentrations of RES, and then cell viability was analyzed every 15 min time intervals using the xCELLigence RTCA DP Instrument (Roche Diagnostics GmbH, Germany). As shown in Fig. 4e, RES significantly suppressed cell proliferation in RCC cells in a time-dependent manner.

### RES inhibits the invasion of RCC cells

Cyclin D1 and VEGF, all regulated by STAT3, are major players in tumor metastasis and has been shown to mediate tumor invasion [49–51]. Whether RES can modulate tumor cell invasion activity was investigated in vitro. To determine this, Caki-1 and 786-O cells were analyzed by Boyden chamber assay. We found that these cells exhibited a very high invasive potential through a thick layer of Matrigel, and this invasive ability was significantly attenuated by RES (Fig. 4f).

### RES enhances the effect of sorafenib on induction of apoptosis in 786-O cells

**Fig. 5:**
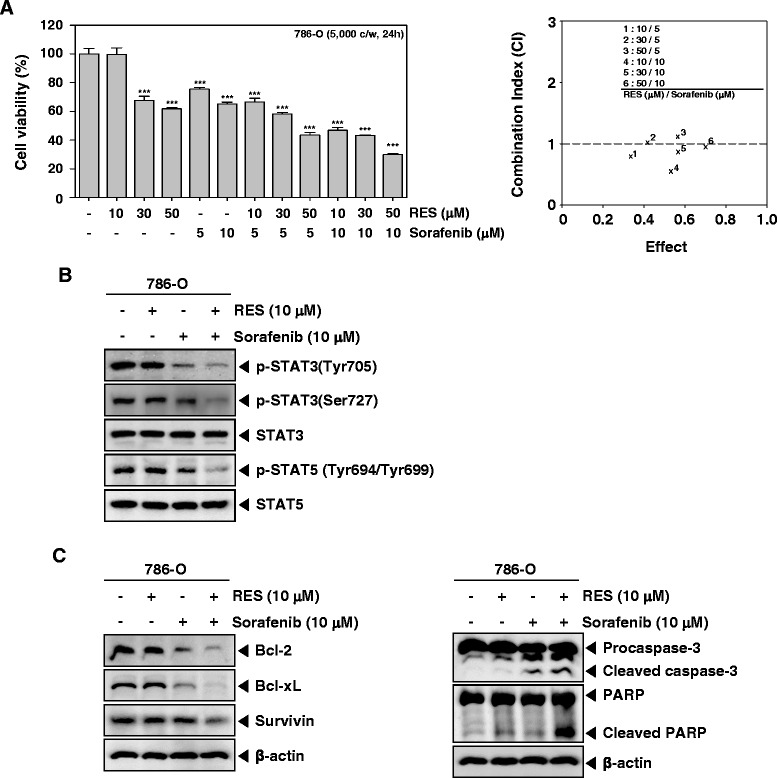
RES potentiates the cytotoxic and apoptotic effects of sorafenib in renal cell carcinoma. **a** and **b** Caki-1 and 786-O cells (5× 10^3^ cells/well) were treated with indicated concentration of RES, and sorafenib for 24 h. The cytotoxicity was determined by MTT assays (*left panel*). The combination index (CI) values were obtained using BiosoftCalcuSyn software (Biosoft, Cambridge, UK) (*right panel*). **c** Caki-1 and 786-O cells (1 × 10^6^ cells/well) were treated with indicated concentration of RES, and sorafenibfor6h. After which whole-cell extracts were prepared and 15 μg portions of those extracts were resolved on 8 % SDS-PAGE gel, electrotransferred onto nitrocellulose membranes, and probed against p-STAT3(Tyr705), p-STAT3(Ser727), STAT3, p-STAT5(Tyr694/699), and STAT5. The results shown here are representative of three independent experiments. **d** and **e** Caki-1 and 786-O cells (1 × 10^6^ cells/well) were treated with indicated concentration of RES, and sorafenibfor24h. Whole-cell extracts were prepared, and 20 μg of the whole-cell lysate was resolved by SDS-PAGE, electrotransferred to nitrocellulose membrane, and then probed with antibodies against bcl-2, bcl-xL, survivin, caspase-3, and PARP. The same blots were stripped and reprobed with β-actin antibody to verify equal protein loading

Sorafenib (a kinase inhibitor) has been used for treating renal cell carcinoma patients. To determine whether RES potentiates the apoptotic effect of sorafenib, we treated 786-O cells with RES combined with sorafenib, and then examined the cell viability with a MTT assay. We found that RES indeed enhanced the cytotoxic effects of sorafenib using CalcuSynsoftware (BIOSOFT, Ferguson, MO) (Fig. 5a). Also, we further examined whether RES can potentiate the inhibitory effect of sorafenib on constitutive STAT3 and STAT5 phosphorylation in 786-O cells by Western blot analysis. As shown in Fig. 5b, RES substantially enhanced the inhibitory effects of sorafenib on phosphorylated STAT3 and STAT5 in 786-O cells. Interestingly, RES or sorafenib alone at sub-optimal concentrations had little effect on levels of bcl-2, bcl-xl, and surviving proteins in 786-O cells. However, treatment of cells with the combination of RES and sorafenib resulted in a marked attenuation in the expression levels of all of these proteins (Fig. 4c). Caspase-3 and PARP cleavage were further increased by the co-treatment of RES along with sorafenib rather than individual agents alone in 786-O cells (Fig. 5d). Overall, these results indicate that RES can significantly enhance the apoptotic effect of sorafenib in 786-O cells through the downregulation of various cell survival proteins.

## Discussion

Although RES has been reported to suppress the proliferation of a wide variety of tumor cell types and induce apoptosis, the mechanism is not yet fully understood in renal cell carcinoma. The aim of the present study was to determine the effect of RES on the STAT3 and STAT5 signaling pathways in two kinds of RCC cells. We found that this polyphenol phytoalexin suppressed constitutive STAT3 (tyrosine residue 705 and serine residue 727) and STAT5 (tyrosine residue 694 and 699) activation in RCC in parallel with the inhibition of JAK1, JAK2, and c-Src activation. We further found that RES induced the expression of protein tyrosine phosphatases such as PTPε and SHP-2 in Caki-1 and 786-O cells, respectively. RES down-regulated the expression of various oncogenic genes, caused the inhibition of proliferation, increased accumulation of cells in S phase, suppressed invasive and colony formation activity, and significantly potentiated the apoptotic effects of sorafenib in RCC cells.

We found for the first time that RES could suppress constitutive STAT3 phosphorylation both at Tyr705 and Ser727, in Caki-1 and 786-O cells. Furthermore, RES had effect on STAT5 phosphorylation at Tyr 694/Tyr 699 residue. We also observed that RES suppressed DNA-binding activity and nuclear translocation of both STAT3 and STAT5. We also observed that RES inhibited the activation of constitutively active JAK1, JAK2, and Src kinases in RCC cells. JAKs are essential for the tyrosine phosphorylation of STAT3/5 in response to growth factors and cytokines [52]. STAT3 and STAT5 phosphorylation clearly plays a pivotal role in the proliferation and survival of a wide variety of tumor cells and that blockade of JAK/STATs signals can provide a potent therapeutic strategy for RCC [13, 34, 35, 53].

We also found evidence that the RES-induced inhibition of STAT3 activation involves a protein tyrosine phosphatase (PTP). Various PTPs have been shown to play an important role in STAT3 signaling, including SHP-1 [54], SHP-2 [55], T-cell PTP [56], PTEN [57], PTP-1D [58], CD45 [59], PTPε [60], and low molecular weight PTP [61, 62].

Indeed, we found for the first time that RES stimulates the expression of PTPε proteins and mRNAs in Caki-1 cells, and induces the expression of SHP-2 proteins and mRNAs in 786-O cells, which correlated with down-regulation of constitutive STAT3/JAK1/2 and Src phosphorylation in these cell lines. Transfection with PTPε and SHP-2siRNA reversed the STAT3 inhibitory effect of RES, thereby further implicating a critical role of these two phosphatases in RES-induced down-regulation of STAT3 signaling cascade.

We further observed that RES can suppress the expression of several STAT3/5-regulated genes; including antiapoptotic gene products (bcl-xl, bcl-2, IAP-1/2, and survivin), cell proliferation (COX-2), inducers of angiogenesis (MMP-9 and VEGF), and cell-cycle regulators (cyclin D1, and cyclin E). Activation of constitutive STAT3/5 can affect to oncogenesis by protecting cancer cells from apoptosis; this implies that suppression of STAT3 activation by agents such as RES could facilitate apoptosis.

The down-regulation of the expression of bcl-2, bcl-xl, and survivin is likely linked with the ability of RES to induce apoptosis in RCC. Inhibition of MMP-9 expression by RES correlated with its observed significant anti-invasive effects in the cells. Recently, sorafenib (a kinase inhibitor) has been approved for treating renal cell carcinoma patients [63]. We further found that RES can significantly enhance the apoptotic effects of sorafenib in RCC cells through the downregulation of various STAT3-regulated proteins and hence could also be used in conjunction with existing anti-RCC therapies.

## Conclusions

Overall, our experimental findings clearly indicate that the pro-apoptotic and anti-invasive effects of RES mediated through the suppression of STAT3/5 activation cascade and its-regulated oncogenic gene products in RCC. Taken together, these results may provide new mechanistic insights into the anti-cancer actions of this phytoalexin.

## Abbreviations

BSA: bovine serum albumin
COX-2: cyclooxygenase-2
HCC: hepatocellular carcinoma
JAK: janus activated kinase
MM: multiple myeloma
MMP-9: matrix metallopeptidase 9
MTT: 3-(4,5-dimethylthiazol-2-yl)-2,5-diphenyltetrazolium bromide
PI: propidium iodide
PTP: protein tyrosine phosphatase
RCC: renal cell carcinoma
SDS: sodium dodecylsulfate
SHP: SH2-containing a ubiquitously expressed tyrosine-specific protein phosphatase
STAT: signal transducers and activators of transcription
TMRE: tetramethylrhodamine, ethyl ester
VEGF: vascular endothelial growth factor
